# Phenotypic vs. genetic cascade screening for familial hypercholesterolemia: A case report

**DOI:** 10.3389/fcvm.2022.982607

**Published:** 2022-08-25

**Authors:** Anastasia V. Blokhina, Alexandra I. Ershova, Alexey N. Meshkov, Anna V. Kiseleva, Marina V. Klimushina, Anastasia A. Zharikova, Evgeniia A. Sotnikova, Vasily E. Ramensky, Oxana M. Drapkina

**Affiliations:** ^1^Laboratory of Clinomics, National Medical Research Center for Therapy and Preventive Medicine of the Ministry of Healthcare of the Russian Federation, Moscow, Russia; ^2^Laboratory of Molecular Genetics, National Medical Research Center for Therapy and Preventive Medicine of the Ministry of Healthcare of the Russian Federation, Moscow, Russia; ^3^Department for the Study of Biochemical Risk Markers of Chronic Noncommunicable Diseases, National Medical Research Center for Therapy and Preventive Medicine of the Ministry of Healthcare of the Russian Federation, Moscow, Russia; ^4^Faculty of Bioengineering and Bioinformatics, Lomonosov Moscow State University, Moscow, Russia; ^5^Laboratory of Genomic and Medical Bioinformatics, National Medical Research Center for Therapy and Preventive Medicine of the Ministry of Healthcare of the Russian Federation, Moscow, Russia; ^6^Department of Fundamental and Applied Aspects of Obesity, National Medical Research Center for Therapy and Preventive Medicine of the Ministry of Healthcare of the Russian Federation, Moscow, Russia

**Keywords:** familial hypercholesterolemia, cascade screening, phenotypic screening, genetic screening, molecular genetic testing, LDLR, duplication

## Abstract

One of the most common autosomal dominant disorders is familial hypercholesterolemia (FH), causing premature atherosclerotic cardiovascular diseases and a high risk of death due to lifelong exposure to elevated low-density lipoprotein cholesterol (LDL-C) levels. FH has a proven arsenal of treatments and the opportunity for genetic diagnosis. Despite this, FH remains largely underdiagnosed worldwide. Cascade screening is a cost-effective method for the identification of new patients with FH and the prevention of cardiovascular diseases. It is usually based only on clinical data. We describe a 48-year-old index patient with a very high LDL-C level without controlled guidelines-based medication, premature atherosclerosis, and a rare variant in the low-density lipoprotein receptor (*LDLR*) gene. Phenotypic cascade screening identified three additional FH relatives, namely the proband's daughter, and two young grandsons. The genetic screening made it possible to rule out FH in the proband's younger grandson. This clinical case demonstrates that genetic cascade screening is the most effective way of identifying new FH cases. We also first described in detail the phenotype of patients with a likely pathogenic variant *LDLR*-p.K223_D227dup.

## Introduction

One of the most common autosomal dominant disorders is familial hypercholesterolemia (FH) (1–3), causing premature atherosclerotic cardiovascular diseases and a high risk of death due to lifelong exposure to elevated low-density lipoprotein cholesterol (LDL-C) levels (4). The genetic basis of FH is well understood and in the vast majority of cases are variants in one of the three genes: low-density lipoprotein receptor gene (*LDLR*), apolipoprotein B gene (*APOB*), and proprotein convertase subtilisin/kexin type 9 gene (*PCSK9*) (1). Highly effective lipid-lowering therapy (LLT) and extracorporeal methods of FH treatment (apheresis) are currently available (4). Despite this, FH remains largely underdiagnosed worldwide (5).

Cascade screening is a mechanism for identifying people with a genetic condition by the process of systematic family members examination using phenotypic or genetic strategy (6). It is a cost-effective method for the identification of new patients with FH and the prevention of cardiovascular disease, as highlighted in the major international guidelines (4–7). Genetic testing is recommended in international guidelines to confirm the FH, but it is not obligated and the diagnosis of FH is usually based only on clinical data (the phenotypic strategy) (4–8). However, if the FH can be diagnosed by clinical data, what are the benefits of genetic testing?

Here, we demonstrate the role of genetic screening for FH in real clinical practice and describe a case of a 48-year-old index patient with premature atherosclerosis and a very high LDL-C level without controlled guidelines-based medication. Genetic testing identified a rare likely pathogenic *LDLR* variant. Phenotypic and then genetic cascade screening confirmed heterozygous FH (HeFH) in proband's daughter with the same *LDLR* variant. Phenotypic screening suggested HeFH in two proband's grandsons, whereas the genetic screening made it possible to rule out FH in the younger grandson. We also first described in detail the phenotype of patients with a likely pathogenic variant *LDLR*: hg19::chr19:11216249_11216263dup, or NM_000527.5:c.667_681dup, NP_000518.1:p.K223_D227dup at the protein level. We observed segregation of this variant in three generations.

## Case description

### Materials and methods

#### Subjects

Three-generation family (a 48-year-old woman (the index patient), the proband's pregnant 30-year-old daughter (gestational age −3.5 weeks), and two grandsons (ten and four-year-old) presented to the Lipid Clinic (National Medical Research Center for Therapy and Preventive Medicine, Moscow, Russia) in October 2020.

#### Genetic analyses

Using the QIAamp DNA Blood Mini Kit (Qiagen, Hilden, Germany), DNA extraction was performed. DNA concentration was measured on a Qubit 4.0 (Thermo Fisher Scientific, Waltham, MA, USA). The libraries for the NGS custom panel were done with the SeqCap EZ Prime Choice Library kit (Roche, Basel, Switzerland). This panel included exon sequences of the *LDLR, APOB, PCSK9*, and *LDLRAP1* genes, as well as other genes, associated with lipid metabolism disorders (*ABCA1, ABCG5, ABCG8, ANGPTL3, APOA1, APOA5, APOC2, APOC3, APOE, CETP, GPD1, GPIHBP1, LCAT, LIPC, LIPI, LMF1, LPL, MTTP, SAR1B, STAP1, USF1*). Next-generation sequencing (NGS) was performed on the Nextseq 550 (Illumina, San Diego, CA, USA). All stages of sequencing were carried out according to the manufacturer's protocols.

After the bioinformatic analysis .bam and .vcf files were generated. For clinical interpretation only variants with minor allele frequency (MAF) <0.01% in the Genome Aggregation Database (gnomAD; http://gnomad.broadinstitule.org) were analyzed according to the American College of Medical Genetics and Genomics/Association for Molecular Pathology (ACMG/AMP2015) guidelines and according to the Clinical Genome Resource (ClinGen) guidelines for *LDLR* variant classification (9, 10).

The validation of variants was done by Sanger sequencing on the Applied Biosystem 3,500 Genetic Analyzer (Thermo Fisher Scientific, Waltham, MA, USA).

### Results

#### Phenotypic cascade screening of the proband and first-degree relatives

A 48-year-old woman presented to the Lipid Clinic in October 2020 with severe hypercholesterolemia. The first blood test for total cholesterol (TC) was done in 2014 (the TC value was about 10 mmol/L) and irregular LLT (rosuvastatin 20 mg daily) was started. In February 2020 TC was 11.46 mmol/L (without LLT). The patient continued rosuvastatin 20 mg daily (LDL-C was 7.81 mmol/L). At the time of the visit, the patient's medications included rosuvastatin 20 mg daily and evolocumab 140 mg per 2 weeks (LDL-C was 3.92 mmol/L).

The patient had been smoking for 5 years and also had a history of early menopause. At a physical examination, she had obesity (height 164 cm, weight 84 kg, and body mass index 31.2 kg/m^2^), bilateral Achilles tendons xanthomas, and xanthelasmas, but not corneal arcus. She also had carotid (duplex ultrasound showed 25–30% stenoses of both carotid bifurcations), femoral (20–25% stenoses of left common femoral and left popliteal arteries), and coronary atherosclerosis (the Agatston score was 252.83). The echocardiography showed aortic valve calcification.

The patient's father had two strokes, with the first event occurring at the age of 55, and died of myocardial infarction at the age of 70. Father's TC levels are unknown. Proband's mother is 69-year-old. According to oral communication provided by the proband, her TC is not high, although the lipid profile is not available. TC levels had also not been measured in the patient's 30-year-old daughter and two grandsons (ten and four-year-old).

**Figure 1:**
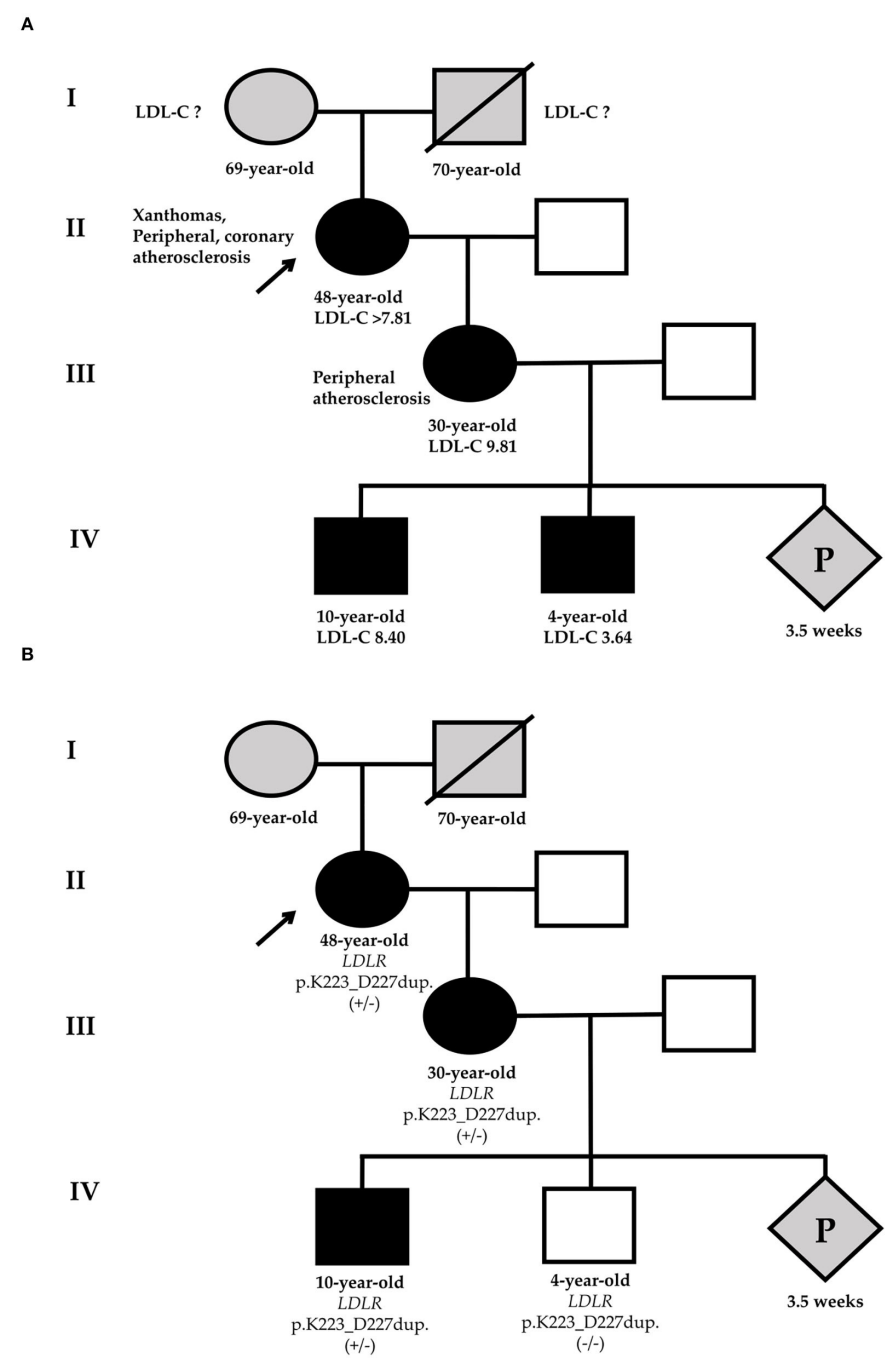
Phenotypic **(A)** and genetic **(B)** cascade screening. According to phenotypic screening, the younger grandson has HeFH [black-filled square **(A)**], in relation to genetic screening, he is healthy [white square **(B)**]. Circles represent females, squares indicate males, and diamond is pregnancy (unknown gender). Black-filled symbols show FH phenotype, gray symbols point to members with an unknown phenotype, and white symbols are healthy family members. Slashes indicate deceased members. +/– or -/- represent heterozygous or wild-type of *LDLR*-p.K223_D227dup variant. The index patient (II-1) is marked with an arrow. LDL-C, low-density lipoprotein cholesterol; P, pregnancy.

Given severe hypercholesterolemia, family history, and examination, the proband has a “definite” diagnosis of HeFH (14 points according to the Dutch Lipid Clinic Network (DLCN)) (11). Thus, HeFH was diagnosed in the proband by clinical data. The presence of FH in a proband indicates the need for phenotypic cascade screening in first-degree relatives. We invited the patient's 30-year-old daughter for examination. She had no xanthomas, xanthelasmas, or corneal arcus, but she had already had peripheral atherosclerosis (duplex ultrasound showed 15–20% stenosis of the right common femoral artery). She also had a high LDL-C level (9.81 mmol/L) but had never taken a LLT (Figure 1A). Therefore, proband's daughter also had a “definite” diagnosis of HeFH (10 points according to the DLCN).

#### Genetic cascade screening of the proband and the proband's daughter

According to the 2019 European Society of Cardiology/European Atherosclerosis Society guidelines for the management of dyslipidaemias, a patient with a clinical FH diagnosis is recommended to undergo molecular genetic testing (recommendations class I, evidence level C) to identify pathogenic or likely pathogenic variants in the *LDLR, APOB*, or *PCSK9* (4).

In the index patient, a total of 89 variants were identified. Of these, only two variants (duplication and missense) had MAF <0.01% and changed the gene amino acid sequence (Table 1).

**Table 1:** Two Two rare variants with MAF <0.01% identified in the proband.

**Gene**	**Genomic coordinates (hg19)**	**Transcript; DNA change; Protein change**	**dbSNP ID**	**gnomAD MAF** **(v. 2.1.1)**	**ACMG interpretation**
*LDLR*	chr19:11216249_11216263dup	NM_000527.5; c.667_681dup; p.K223_D227dup	-	-	Likely pathogenic (PM4, PM2, PP1, PP5)
*ABCG5*	chr2:44059195G>C	NM_022436.3; c.293C>G; p.A98G	rs145164937	0.002253	Variant of uncertain significance (PM2, PP3)

*ACMG, American College of Medical Genetics and Genomics; gnomAD, Genome Aggregation Database; MAF, minor allele frequency*.

One of the variants is a heterozygous missense variant in *ABCG5*: hg19:chr2:44059195G>C, NM_022436.3:c.293C>G, NP_071881.1:p.A98G, rs145164937. According to the ACMG/AMP2015 guidelines, this variant has two evidences of pathogenicity: MAF <0.01% in gnomAD (PM2) and multiple *in silico* predictions as deleterious (PP3). Thus, this variant can be classified as uncertain significance.

**Figure 2:**
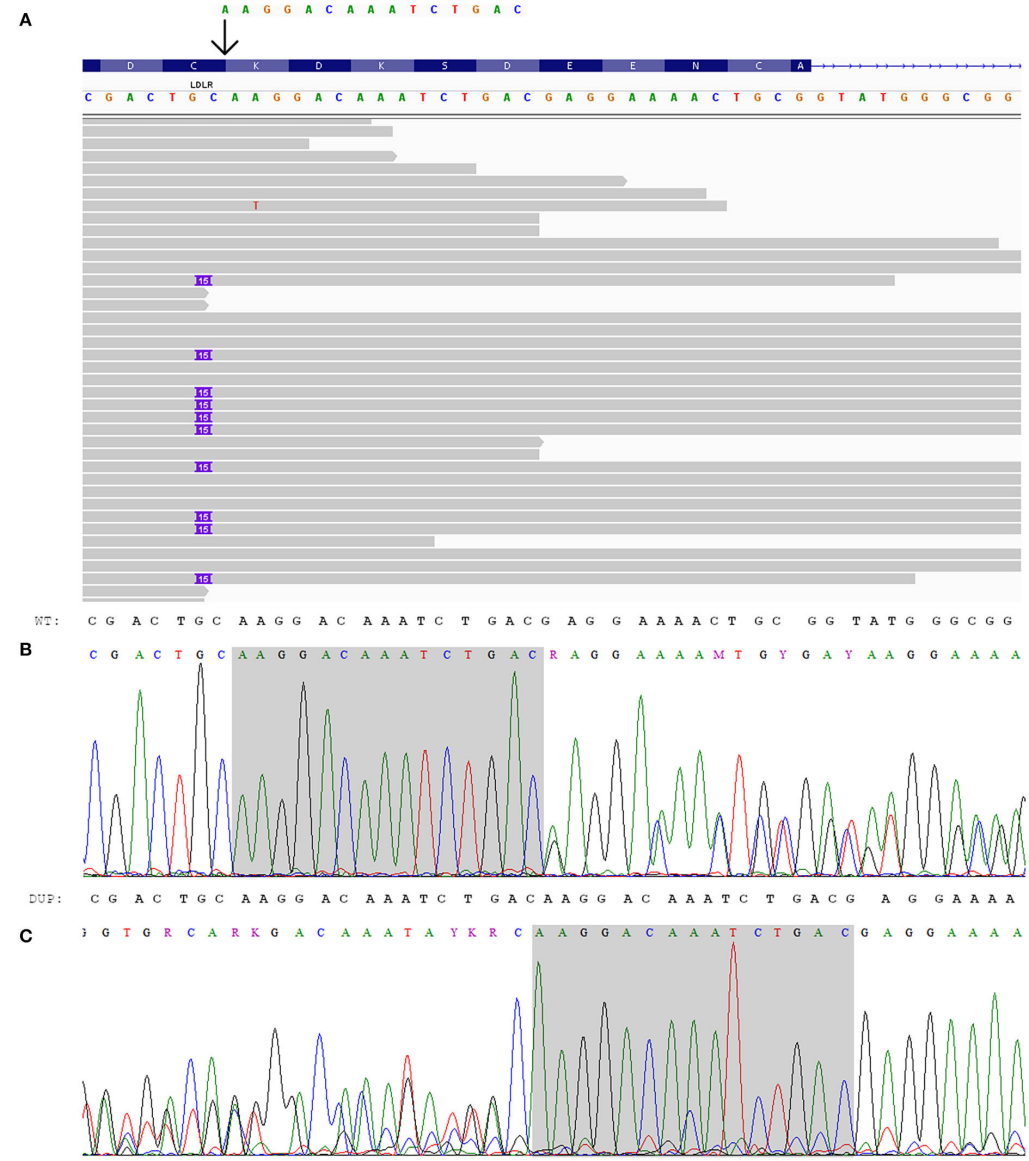
Genetic analysis of the index patient. **(A)** Integrated genome view of *LDLR*-p.K223_D227dup with IGV (12). **(B, C)** Electropherograms of *LDLR*-p.K223_D227dup: **(B)** sequence from the forward primer, **(C)** sequence from the reverse primer (reverse complement).

At the same time, the proband had a heterozygous likely pathogenic duplication in *LDLR*: chr19:11216249_11216263dup, NM_000527.5:c.667_681dup, NP_000518.1:p.K223_D227dup. The validation of this variant was done by Sanger sequencing (Figure 2). This variant was also confirmed in the proband's daughter by Sanger sequencing. The observed criteria of pathogenicity according to the ACMG/AMP2015 are PM4, PM2, PP1, and PP5 (9).

#### Phenotypic cascade screening of the second-degree relatives

Considering the presence of HeFH in the proband's daughter, we analyzed the proband's grandson's lipid profile (Figure 1A).

For the diagnosis of FH in the proband's grandsons, the pediatric diagnostic criteria should be used (13). The eldest grandson has a very high LDL-C level and FH can be diagnosed by clinical data (LDL-C level and family history). The younger grandson's LDL-C level is not very high, but the positive genetic testing of his mother may suggest HeFH (LDL-C level ≥3.5 mmol/L).

#### Genetic cascade screening of the second-degree relatives

The same *LDLR* variant was verified in elder proband's grandson by Sanger sequencing. Despite that phenotypic screening suggested HeFH in two proband's grandsons, the genetic screening made it possible to rule out FH in the younger grandson (Figure 1B).

#### Personalized treatment and recommendations

Considering HeFH and additional risk factors, the proband has a very high cardiovascular risk. The LDL-C target level is less than 1.4 mmol/L. After 1 month of high-intensity statin therapy (rosuvastatin 40 mg daily) with ezetimibe 10 mg daily and the injection of evolocumab (140 mg per 2 weeks), she achieved the LDL-C target level (LDL-C 1.29 mmol/L).

The proband's daughter needs high-intensity statin therapy and her LDL-C target level is less than 1.8 mmol/L. However, she is currently pregnant. LLT should not be given during pregnancy or during the breastfeeding period (4). Therefore, we are going to initiate the LLT after the end of breastfeeding.

The proband's elder grandson was sent for further examination to a specialized pediatric department. He should be educated to adopt a proper diet and treated with a statin. His LDL-C target should be <3.5 mmol/L (4, 13).

We recommended a lipid-lowering diet and dynamic monitoring of LDL-C level for the proband's younger grandson. For future perspectives, NGS of the lipid metabolism disorder genes and determination of polygenic risk scores for LDL-C level may be considered. Finally, we recommended screening for HeFH of the future child of the proband's daughter after his birth.

## Discussion

Familial hypercholesterolemia is one of the most common monogenic disorders with underestimated real prevalence. The meta-analyses of 2020 showed that the prevalence of HeFH in the general population is one in 313 (14). A recent population study in Russia showed the prevalence of HeFH is one in 173 (15).

In patients with FH cumulative LDL-C exposure is the leading cause of premature cardiovascular events (16). Hence, early identification of FH and its treatment with highly effective and safe LLT is necessary. Nevertheless, despite the high prevalence, the presence of diagnostic criteria, as well as the availability of NGS, and effective methods for reducing LDL-C levels, FH remains underdiagnosed and undertreated worldwide (5).

Here, we presented a three-generation family, where we identified HeFH by both phenotypic and genetic cascade screening in the proband's family members.

Using phenotypic cascade screening we confirmed HeFH by clinical data at the proband, the proband's daughter, and grandsons. The younger grandson's LDL-C level is not very high, but based on the positive genetic testing of his mother we may suggest HeFH. However, the genetic screening allowed to rule out FH in the younger grandson. Given FH at the proband's daughter, her future child also has a 50% probability of inheriting FH.

Using NGS we identified a rare *LDLR* duplication variant p.K223_D227dup with segregation in two proband's relatives. According to the ClinGen 2021 guidelines for *LDLR* variant classification, the duplication meets two moderate criteria, such as absence in the gnomAD (PM2) and segregation with phenotype in three informative meioses (PP1_Moderate). Besides, this variant was found in two unrelated FH cases (PS4_Supporting) (17) and also identified in a patient with FH diagnosis based on validated clinical criteria, after alternative causes of high cholesterol, were excluded (PP4). Therefore, p.K223_D227dup can be classified as likely pathogenic. This variant has not been previously described in patients with FH in the European population (18). The identification and interpretation of pathogenicity of new or rare FH-associated variants supplement the knowledge about the spectrum of FH variants. We also first described in detail the phenotype of patients with p.K223_D227dup based on segregation in three generations. This made it possible to increase the segregation level of pathogenicity from supporting to moderate.

Cascade screening is the step-by-step identification of patients with a monogenic disease among the proband's first-, second-, and, when possible, third-degree relatives. There are two strategies for cascade screening of the proband's relatives: phenotypic and genetic (6).

Considering a phenotypic strategy, FH can be based on clinical data, using the DLCN criteria (11), the Simon Broome Register Diagnostic Criteria (19), the biochemical criteria for diagnosis of the proband's relatives (8), and the criteria of FH in children (13). Genetic testing is also recommended in international guidelines to confirm the FH and conduct cascade screening (4–8). However, if the FH can be diagnosed by clinical data, what are the benefits of genetic testing?

Firstly, genetic testing helps to confirm the genetic basis of the disease and clarify the cardiovascular risk stratification. In this case, the positive genetic testing results for the proband, the proband's daughter, and especially for the proband's elder grandson may suggest a greater risk of developing cardiovascular events compared with a clinical FH phenotype without an established monogenic etiology. This statement has been noted in a number of studies (20, 21). Furthermore, the gene type and even the pathogenic variant type can affect an individual's LDL-C level and consequently the risk of developing premature coronary and peripheral atherosclerosis (7). In a number of studies, it has been noted that *LDLR* pathogenic variants carriers have a greater level of LDL-C compared to *APOB* or *PCSK9* variants (22). In this case, all positive *LDLR* variant patients had a very high LDL-C level. The LDL-C level of the 10-year-old male was almost comparable to his 30-year-old mother.

Besides this, cardiovascular risk in patients with FH is considered manageable. Using genetic testing not only does not decrease perceptions of control over FH, cholesterol levels, or cardiovascular diseases but on the contrary, leads to more positive patient perceptions of FH (23, 24). It means that patients feel personally responsible for managing their health. Patients consider that FH genetic testing is very important and, regardless of the result, are ready to recommend genetic screening to their family members, which, in turn, can help to improve FH diagnosis (24). In this case, all available proband's relatives underwent genetic screening.

A meta-analysis of 17 studies performed in 2016 did not show any significant behavioral changes in smoking, physical activity, or dietary intake before and after the genetic testing. However, only one study of patients with FH was included in this meta-analysis (25). On the other hand, several studies of patients with FH showed other behavioral changes, such as changes in the perception of the most effective way to achieve LDL-C level. The patients with pathogenic variants consider their disease more accurately, with increased motivation to take LLT (23, 24). In this work after the positive genetic testing results, the proband began to take all prescribed LLT and achieved LDL-C targets. The early diagnosis is associated with appropriate treatment, lifestyle modifications, and in turn better prognosis. However, children have a silent presentation of FH and phenotypic screening alone is not enough. For examination of children and adolescents, it is not often possible to identify such phenotypic markers as xanthomas, corneal arcus, arteries atherosclerotic changes, or premature cardiovascular events (13, 26).

Genetic testing in childhood and adolescence, in a period of the formation of healthy lifestyle habits, is essential for ensuring long-term adherence and can contribute to the primary prevention of cardiovascular diseases. Starting FH treatment from a young age, and being surrounded by other family members during treatment, facilitates adherence and has particular importance in the treatment of FH (27). Children with FH variant are more understanding of their genetic disease basis. Furthermore, FH carrier children demonstrate high feelings of control over their disease (28).

**Figure 3:**
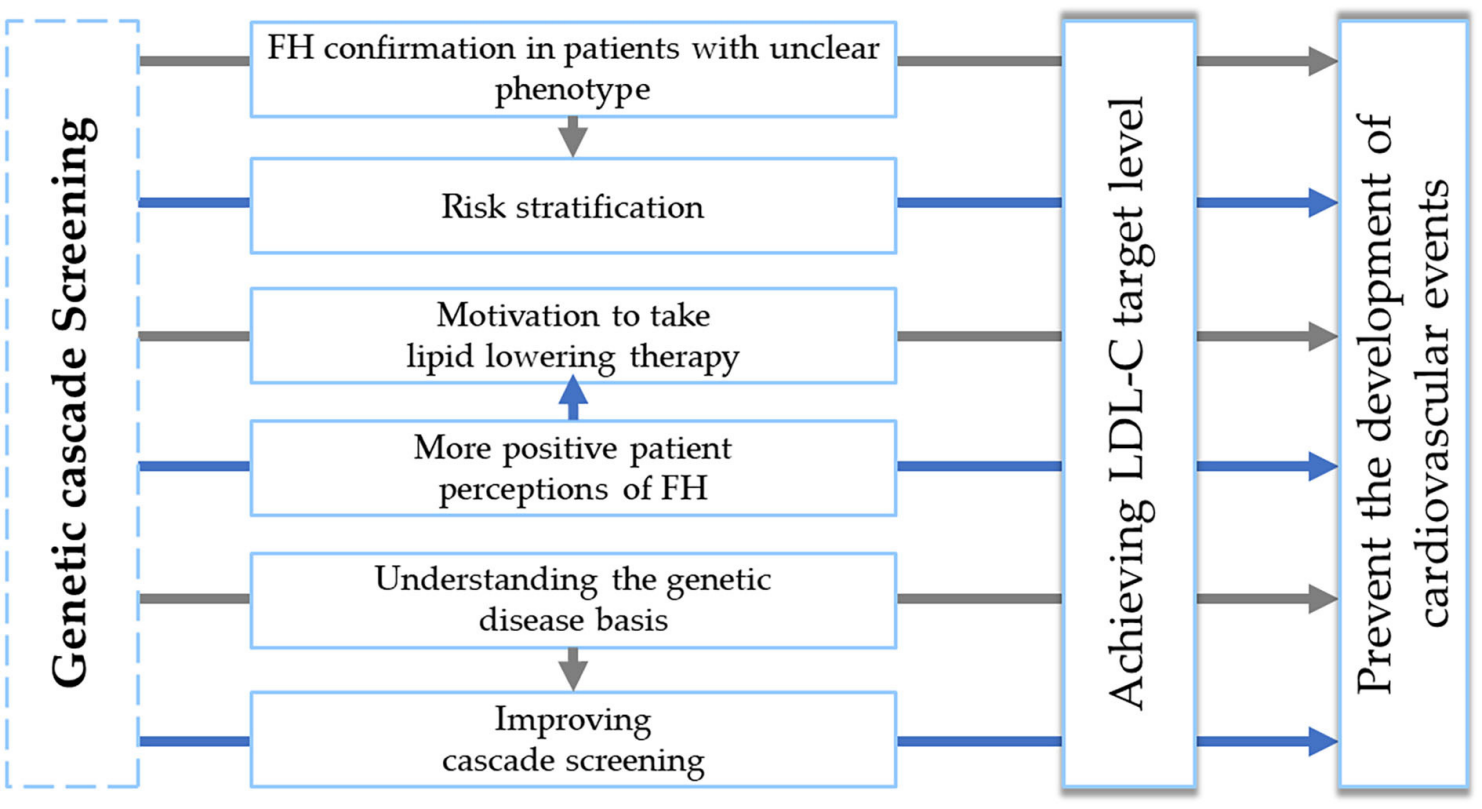
Genetic cascade screening benefits. FH, familial hypercholesterolemia. LDL-C, low-density lipoprotein cholesterol.

Characterization of genetic cascade screening benefits is presented in Figure 3.

It is important to note that FH variants are verified only in 20–80% of patients with HeFH phenotype and at the same time the negative genetic testing results do not rule out a diagnosis of FH (29, 30). In this case, the genetic screening allowed to exclude the presence of *LDLR* variant p.K223_D227dup in the younger grandson. It is likely that his elevated LDL-C level is due to diet. Even with a negative FH genetic analysis, it is always necessary to remember about other lipid metabolism genetic disorders or that some patients with the typical FH phenotype may have a polygenic etiology of hypercholesterolemia (31). Targeted, exome or even genome sequencing, as well as genetic risk scores, may help to establish the reason for hypercholesterolemia. This highlights the unique role of genetic testing in the personalized medicine era.

## Conclusion

In summary, given the high FH prevalence, the availability of NGS, and effective methods for reducing LDL-C levels, genetic cascade screening is the most reliable, effective, and expedient method of personalized medicine for identifying new FH cases, including children and adolescents at the preclinical stage. This helps to clarify the cardiovascular risk stratification, and carry out targeted preventive measures, including lifestyle, cardiovascular risk factors correction, and early initiation of intensive LLT to achieve LDL-C targets and prevent the development of cardiovascular events. In unclear phenotypes, especially in children, genetic screening may help to rule out FH.

## Data availability statement

The datasets presented in this study can be found in online repositories. The names of the repository/repositories and accession number(s) can be found below: https://www.ncbi.nlm.nih.gov/, SRR19880502.

## Ethics statement

Ethical review and approval was not required for the study on human participants in accordance with the local legislation and institutional requirements. The patients/participants provided their written informed consent to participate in this study. Written informed consent was obtained from the individual(s), and minor(s)' legal guardian/next of kin, for the publication of any potentially identifiable images or data included in this article.

## Author contributions

AB was treating the patients, performing their follow-ups, carrying out the interpretation of NGS data, and writing this case. AE was treating the patients, supervising all parts of this article's preparation, and editing the article. AM supervised the genetic testing of the patient and provided valuable comments on this case. AK performed the NGS and edited this article. MK performed the Sanger sequencing. AZ carried out the bioinformatic analyses of NGS data. ES performed the NGS. VR supervised the bioinformatic analyses of NGS data and edited this article. OD is the chief of the center who provided valuable comments on this case. All authors contributed to the article and approved the submitted version.

## Conflict of interest

The authors declare that the research was conducted in the absence of any commercial or financial relationships that could be construed as a potential conflict of interest.

## Publisher's note

All claims expressed in this article are solely those of the authors and do not necessarily represent those of their affiliated organizations, or those of the publisher, the editors and the reviewers. Any product that may be evaluated in this article, or claim that may be made by its manufacturer, is not guaranteed or endorsed by the publisher.

## References

[1] Berberich AJ Hegele RA. The complex molecular genetics of familial hypercholesterolaemia. Nat Rev Cardiol. (2019) 16:9–20. 10.1038/s41569-018-0052-6 29973710

[2] Hu P Dharmayat KI Stevens CA Sharabiani MT Jones RS Watts GF. Prevalence of familial hypercholesterolemia among the general population and patients with atherosclerotic cardiovascular disease: a systematic review and meta-analysis. Circulation. (2020) 141:1742–59. 10.1161/CIRCULATIONAHA.119.044795 32468833

[3] Toft-Nielsen F Emanuelsson F Benn M. Familial hypercholesterolemia prevalence among ethnicities-systematic review and meta-analysis. Front Genet. (2022) 13:840797–840797. 10.3389/fgene.2022.840797 35186049 PMC8850281

[4] Mach F Baigent C Catapano AL Koskinas KC Casula M Badimon L. 2019 ESC/EAS guidelines for the management of dyslipidaemias: lipid modification to reduce cardiovascular risk: the task force for the management of dyslipidaemias of the European Society of Cardiology (ESC) and European Atherosclerosis Society (EAS). Eur Heart J. (2020) 41:111–88. 10.1093/eurheartj/ehz455 31504418

[5] Nordestgaard BG Chapman JM Humphries SE Ginsberg HN Masana L Descamps OS. Familial hypercholesterolaemia is underdiagnosed and undertreated in the general population: guidance for clinicians to prevent coronary heart disease: consensus statement of the European Atherosclerosis Society. Eur Heart J. (2013) 34:3478–3490. 10.1093/eurheartj/eht273 23956253 PMC3844152

[6] Harada-Shiba M Arai H Ishigaki Y Ishibashi S Okamura T Ogura M. Guidelines for diagnosis and treatment of familial hypercholesterolemia 2017. J Atheroscler Thromb. (2018) 25:751–70. 10.5551/jat.CR003 29877295 PMC6099072

[7] Sturm AC Knowles JW Gidding SS Ahmad ZS Ahmed CD Ballantyne CM. Clinical genetic testing for familial hypercholesterolemia: JACC scientific expert panel. J Am Coll Cardiol. (2018) 72:662–80. 10.1016/j.jacc.2018.05.044 30071997

[8] DeMott K Nherera L Shaw EJ Minhas R Humphries SE Kathoria M. Clinical Guidelines and Evidence Review for Familial Hypercholesterolaemia: the Identification and Management of Adults and Children with Familial Hypercholesterolaemia. London: National Collaborating Centre for Primary Care and Royal College of General Practitioners (2008). 14 p.

[9] Richards S Aziz N Bale S Bick D Das S Gastier-Foster J. Standards and guidelines for the interpretation of sequence variants: a joint consensus recommendation of the American College of Medical Genetics and Genomics and the Association for Molecular Pathology. Genet Med. (2015) 17:405–23. 10.1038/gim.2015.30 25741868 PMC4544753

[10] Chora JR Iacocca MA Tichy L Wand H Kurtz CL Zimmermann H. The clinical genome resource (ClinGen) familial hypercholesterolemia variant curation expert panel consensus guidelines for LDLR variant classification. Genet Med. (2022) 24:293–306. 10.1016/j.gim.2021.09.012 34906454 PMC12558601

[11] Civeira F. Guidelines for the diagnosis and management of heterozygous familial hypercholesterolemia. Atherosclerosis. (2004) 173:55–68. 10.1016/j.atherosclerosis.2003.11.010 15177124

[12] Robinson JT Thorvaldsdóttir H Wenger AM Zehir A Mesirov JP. Variant review with the integrative genomics viewer. Cancer Res. (2017) 77:e31–4. 10.1158/0008-5472.CAN-17-0337 29092934 PMC5678989

[13] Wiegman A Gidding SS Watts GF Chapman JM Ginsberg HN Cuchel M. Familial hypercholesterolaemia in children and adolescents: gaining decades of life by optimizing detection and treatment. Eur Heart J. (2015) 36:2425–2437. 10.1093/eurheartj/ehv157 26009596 PMC4576143

[14] Beheshti SO Madsen CM Varbo A Nordestgaard BG. Worldwide prevalence of familial hypercholesterolemia: meta-analyses of 11 million subjects. J Am Coll Cardiol. (2020) 75:2553–2566. 10.1016/j.jacc.2020.03.057 32439005

[15] Meshkov AN Ershova AI Kiseleva AV Shalnova SA Drapkina OM Boytsov SA. The prevalence of heterozygous familial hypercholesterolemia in selected regions of the Russian federation: the FH-ESSE-RF study. J Pers Med. (2021) 11:464. 10.3390/jpm11060464 34074024 PMC8225162

[16] Tada H Okada H Nohara A Yamagishi M Takamura M Kawashiri MA. Effect of cumulative exposure to low-density lipoprotein-cholesterol on cardiovascular events in patients with familial hypercholesterolemia. Circ J. (2021) 28:CJ-21. 10.1093/eurheartj/ehab724.2555 34011825

[17] Miyake Y Yamamura T Sakai N Miyata T Kokubo Y Yamamoto A. Update of Japanese common LDLR gene mutations and their phenotypes: mild type mutation L547V might predominate in the Japanese population. Atherosclerosis. (2009) 203:153–60. 10.1016/j.atherosclerosis.2008.07.005 18718593

[18] Meshkov A Ershova A Kiseleva A Zotova E Sotnikova E Petukhova A. The LDLR, APOB, and PCSK9 variants of index patients with familial hypercholesterolemia in Russia. Genes. (2021) 12:66. 10.3390/genes12010066 33418990 PMC7825309

[19] Simon Broome Register Group. Risk of fatal coronary heart disease in familial hypercholesterolaemia. BMJ. (1991) 303:893–6. 1933004 10.1136/bmj.303.6807.893 PMC1671226

[20] Khera AV Won HH Peloso GM Lawson KS Bartz TM Deng X. Diagnostic yield and clinical utility of sequencing familial hypercholesterolemia genes in patients with severe hypercholesterolemia. J Am Coll Cardiol. (2016) 67:2578–89. 10.1016/j.jacc.2016.03.520 27050191 PMC5405769

[21] Tada H Kawashiri MA Nohara A Inazu A Mabuchi H Yamagishi M. Impact of clinical signs and genetic diagnosis of familial hypercholesterolaemia on the prevalence of coronary artery disease in patients with severe hypercholesterolaemia. Eur Heart J. (2017) 38:1573–9. 10.1093/eurheartj/ehx004 28159968

[22] Doi T Hori M Harada-Shiba M Kataoka Y Onozuka D Nishimura K. Patients with LDLR and PCSK9 gene variants experienced higher incidence of cardiovascular outcomes in heterozygous familial hypercholesterolemia. J Am Heart Assoc. (2021) 10:e018263. 10.1161/JAHA.120.018263 33533259 PMC7955325

[23] Marteau T Senior V Humphries SE Bobrow M Cranston T Crook MA. Psychological impact of genetic testing for familial hypercholesterolemia within a previously aware population: a randomized controlled trial. A J Med Genet A. (2004) 128:285–93. 10.1002/ajmg.a.30102 15216550

[24] Marchand M Chen V Trinder M Cermakova L Brunham LR. Patient perspectives regarding genetic testing for familial hypercholesterolemia. CJC Open. (2021) 3:557–64. 10.1016/j.cjco.2020.12.006 34027362 PMC8134866

[25] Holland GJ French DP Griffin SJ Prevost AT Sutton S King S. The impact of communicating genetic risks of disease on risk-reducing health behaviour: systematic review with meta-analysis. BMJ. (2016) 352:i1102. 10.1136/bmj.i1102 26979548 PMC4793156

[26] Tada H Takamura M Kawashiri MA. Familial hypercholesterolemia: a narrative review on diagnosis and management strategies for children and adolescents. Vascul Health Risk Manag. (2021) 17:59. 10.2147/VHRM.S266249 33628029 PMC7898200

[27] Kinnear FJ Wainwright E Perry R Lithander FE Bayly G Huntley A. Enablers and barriers to treatment adherence in heterozygous familial hypercholesterolaemia: a qualitative evidence synthesis. BMJ Open. (2019) 9:e030290. 10.1136/bmjopen-2019-030290 31371299 PMC6677970

[28] Meulenkamp TM Tibben A Mollema ED Van Langen IM Wiegman A De Wert GM. Predictive genetic testing for cardiovascular diseases: impact on carrier children. A J Med Genet A. (2008) 146:3136–46. 10.1002/ajmg.a.32592 19012345

[29] Talmud PJ Shah S Whittall R Futema M Howard P Cooper JA. Use of low-density lipoprotein cholesterol gene score to distinguish patients with polygenic and monogenic familial hypercholesterolaemia: a case-control study. Lancet. (2013) 381:1293–301. 10.1016/S0140-6736(12)62127-8 23433573

[30] Trinder M Li X DeCastro ML Cermakova L Sadananda S Jackson LM. Risk of premature atherosclerotic disease in patients with monogenic versus polygenic familial hypercholesterolemia. J Am Coll Cardiol. (2019) 74:512–22. 10.1016/j.jacc.2019.05.043 31345425

[31] Vrablik M Tichý L Freiberger T Blaha V Satny M Hubacek JA. Genetics of familial hypercholesterolemia: new insights. Front Genet. (2020) 11:574474. 10.3389/fgene.2020.574474 33133164 PMC7575810

